# Mechanisms of psilocybin on the treatment of posttraumatic stress disorder

**DOI:** 10.1177/02698811241286771

**Published:** 2024-10-03

**Authors:** Charles Choi, Danica E Johnson, David Chen-Li, Joshua Rosenblat

**Affiliations:** 1 Department of Psychiatry, University of Toronto, Toronto, ON, Canada; 2 Mood Disorders Psychopharmacology Unit, Poul Hansen Depression Center, University Health Network, Toronto, ON, Canada; 3 Institute of Medical Science, University of Toronto, Toronto, ON, Canada

**Keywords:** Psilocybin, posttraumatic stress disorder, psychedelics, PTSD

## Abstract

Posttraumatic stress disorder (PTSD) is a condition that can develop after a traumatic event, causing distressing symptoms, including intrusive re-experiencing symptoms, alterations in mood and cognition, and changes in arousal and reactivity. Few treatment options exist for patients who find conventional psychotherapy and pharmacotherapy to be inaccessible, ineffective, or intolerable. We explore psilocybin as a potential treatment option for PTSD by examining the neurobiology of PTSD as well as psilocybin’s mechanism of action. Based on both pharmacodynamic and psychoanalytic principles, psilocybin may be an underexplored treatment option for patients with PTSD, though further research is required.

## Introduction

### Definition and prevalence of PTSD

Posttraumatic stress disorder (PTSD) is a condition that can develop after direct or indirect exposure to an actual or threatened serious traumatic event, including death, serious injury, or sexual violence. Patients with PTSD often suffer from re-experiencing, which results in them avoiding situations and places reminiscent of traumatic memories. They may also develop negative beliefs about themselves or the world after the traumatic experience. Many experience alterations in arousal and reactivity in the form of irritability, sleep disturbance, hypervigilance, or concentration difficulties (American Psychiatric Association, 2013). The World Health Organization Mental Health Survey estimates a lifetime prevalence of PTSD to be approximately 3.9%, and identified younger age, female gender, unmarried, lower education levels and incomes as risk factors for PTSD (Koenen et al., 2017).

### Pathophysiology of PTSD

The brain regions associated with PTSD include those associated with the acquisition and expression of fear, including the amygdala complex, hippocampus, insular cortex, prefrontal cortex, striatum, thalamus, and sensory areas. Most of the research has centered around the role of the amygdala (Ressler et al., 2022). Axonal projections from neurons within the amygdala to various parts of the nervous system are responsible for reflexes associated with fear. These reflexes include increased startled responses via projections to the reticularis pontis caudalis as well as hypothalamic–pituitary–adrenal activation via projections to the paraventricular nucleus of the hypothalamus. Hippocampal activity is associated with the extinction of cued fear memories, and smaller hippocampal volumes have been noted in patients with chronic PTSD. Activity in the medial prefrontal cortex is also associated with inhibition of threat-related memories and behaviors, and both decreased prefrontal cortex activation and reduced white matter integrity of the uncinate fasciculus connecting the prefrontal cortex to other regions of the brain have been observed in patients with PTSD (Ressler et al., 2022).

In addition to changes in neural pathways, PTSD has been associated with changes in levels of several neurotransmitters, including changes to dopamine, norepinephrine, and serotonin, as highlighted in Table 1 (Sherin and Nemeroff, 2011).

**Table 1.** Neurochemical changes in PTSD and effect of psilocybin on the brain.

Neurotransmitter/brain region	Changes in PTSD	Symptom caused by change	Potential effect of psilocybin
Dopamine	Unclear. Increased urinary excretion of dopamine; possibly increased mesolimbic dopamine activating the HPA axis; changes in metabolism of dopamine remains unclear	Interferes with fear conditioning by mesolimbic system. High levels of dopamine relative to serotonin can lead to impulsive behaviour	Increases levels, believed to cause antidepressant effects
Norepinephrine	Increased levels/activity	Increased arousal, startle response, encoding of fear memories	
		Increased pulse, blood pressure, and response to memories	
Serotonin	Decreased concentration in dorsal and median raphe	Disturbed dynamic between amygdala and hippocampus	Partial agonism, mainly at the 2A receptor
GABA	Decreased activity	Compromised anxiolytic effects	
Glutamate	Increased levels	Increased risk of derealization and dissociation	
Amygdala	Hyperactivity	Activation of fear response and hypothalamic-pituitary-adrenal axis	Reduced amygdala activity, decreased threat response, and increased response to positive stimuli
Hippocampus	Smaller volume	Less extinction of cued fear memories	
Medial prefrontal cortex	Decreased activation of prefrontal cortex and decreased white matter integrity in the uncinate fasciculus (Ressler et al., 2022)	Less inhibition of threat-related memories and behaviours	

### Current treatment options for PTSD

#### Psychotherapy

Evidence-based psychotherapy options for PTSD include Trauma-Focused Cognitive Behavioural Therapy (TF-CBT), Eye Movement Desensitization and Reprocessing (EMDR), and stress management (Katzman et al., 2014). TF-CBT, which includes prolonged exposure therapy as well as cognitive processing therapy, has been the most thoroughly studied (Yehuda et al., 2015). Generally, psychotherapy is delivered through weekly sessions over 6–16 weeks, though patients may require more sessions over months or years. Other nontrauma-focused psychological treatments, including supportive therapy, nondirective counselling, or psychodynamic therapy, are often used, although they do not reduce PTSD symptoms as significantly as trauma-focused therapy (Bisson and Andrew, 2007). In many cases, psychotherapy is not always accessible as patients pay out-of-pocket or must rely on private insurance coverage.

#### Meditation

Emerging data suggest meditation may be helpful for PTSD. A narrative review consisting mainly of randomized controlled trials found various forms of meditation and mindfulness may be effective in improving PTSD symptoms among US veterans (Haider et al., 2021). Meditation and psilocybin exert similar effects on the body and can induce an altered state of consciousness or induce mystical experiences characterized by a sense of unity and interconnectedness (Holas and Kamińska, 2023). Moreover, early data suggest concomitant meditation and psilocybin use may be synergistic (Singer et al., 2024).

#### Pharmacotherapy

Selective Serotonin Reuptake Inhibitors (SSRI), namely fluoxetine, paroxetine, and sertraline, as well as venlafaxine, are generally recommended as first-line pharmacotherapy options for PTSD (American Psychological Association, 2017; Ipser and Stein, 2012). PTSD treatment guidelines vary in how strongly they recommend antipsychotics, though most suggest their use as adjunctive therapy (Yehuda et al., 2015). Guidelines also differ in their recommendation of other medications, including tricyclic antidepressants, mirtazapine, topiramate, and monoamine oxidase inhibitors. Due to the discrepancies in recommendations across different guidelines, clinicians often face difficulty in choosing options for pharmacotherapy. In addition, patients may exhaust the few recommended treatment options which exist when they experience subclinical benefits or intolerable side effects with pharmacological agents. As such, current psychological and pharmacological treatment options can be inaccessible, ineffective, and intolerable, frustrating both clinicians and patients alike. PTSD is also associated with high illness burden, with recurrence rates ranging from 5% to 57% (Brooks and Greenberg, 2024), and a lifetime suicide attempt of 14.3% (Stevens et al., 2013).

## Psychedelics and psilocybin

Psychedelics refers to psychoactive compounds that alter mood, perception, and cognitive processes (Nichols, 2016). Based on their chemical structure, psychedelics can be divided into three categories: tryptamines (e.g., psilocybin and its active metabolite psilocin), ergolines (e.g., lysergic acid), and phenethylamines (e.g., 3,4-methylenedioxymethamphetamine) (Kelmendi et al., 2022). There has been a recent growing interest by researchers and policymakers in the potential role of psychedelics for treating psychiatric conditions.

Psilocybin is a plant alkaloid derived from tryptamine precursors. It is found in a variety of mushroom species and has been used by Indigenous people in Central and South America (Reiff et al., 2020). Although the exact mechanism of psilocybin and psilocin is still under investigation, several mechanisms for the therapeutic benefits of psilocybin have been postulated, including its effect on emotional modulation, neurotransmitters, and neuroplasticity.

### Current uses of psychedelics for PTSD

For treatment of PTSD using psychedelics, studies have focused on the role of MDMA-assisted psychotherapy. A double-blind RCT followed by an open-label crossover study by Mithoefer et al. (2011) reported that 10 out of 12 patients saw clinical improvement (defined as 30% or greater improvement in PTSD symptoms measured 2 months after the second experimental session) in patients assigned to MDMA 125 mg with adjunctive psychotherapy, whereas two out of eight patients assigned to placebo with adjunctive psychotherapy experienced clinical improvement. The open-label crossover component allowed participants initially assigned to the placebo arm to receive MDMA, and clinical response was 100% in these patients. This study is limited by the fact that more additional psychotherapy sessions were provided to subjects in the MDMA arm to provide support integration in those who experienced anxiety and other difficulties.

Mitchell et al. (2021) found significant decreases in PTSD symptoms and functional impairment 18 weeks after baseline measurements among patients who received manualized therapy and were randomized to receive MDMA over those who received manualized therapy and placebo (*n* = 44). These studies, like others investigating psychedelics, are limited by a small sample size, relatively short follow-up period, and difficulty with blinding among participants due to the distinct psychogenic effects of psychedelics.

A pooled analysis of six randomized, double-blind, controlled trials utilizing an active dose of MDMA (75–125 mg) also found large treatment effects (estimated mean difference in Clinician-Administered PTSD Scale (CAPS-5) score of −22.0, SE = 5.17) for MDMA-assisted psychotherapy for treatment of PTSD (Mithoefer et al., 2019). Long-term follow-up of these data at 12 months showed that PTSD symptom improvement continued for at least 12 months posttreatment.

### Current use of psilocybin for mood disorders

The focus of studies on psilocybin thus far has been on the treatment of depression and anxiety rather than on the treatment of PTSD. Carhart-Harris et al. (2021) reported psilocybin 25 mg improved depressive symptoms es as effectively as escitalopram (10–20 mg depending on tolerability experienced by participants) after 6 weeks among patients with moderate-to-severe major depressive disorder. Furthermore, a higher incidence of adverse reactions including anxiety, dry mouth, sexual dysfunction, or emotional blunting was noted in the escitalopram group compared to the psilocybin group. Davis et al. (2021) found depressive symptoms improved in patients receiving psilocybin-assisted psychotherapy when measured between 4 and 8 weeks after baseline compared to patients on the waiting list. Raison et al. (2023) found that psilocybin 25 mg with psychological support led to greater improvement in symptom and disability compared to placebo when measured at day 43 with no reported significant side effects. Current data support the use of psilocybin for PTSD.

Although data are emerging for the use of MDMA for PTSD and psilocybin for mood disorders, only a limited number of studies have examined the potential role of psilocybin on PTSD. Anderson et al. (2020) studied the effect of psilocybin-assisted group psychotherapy in a single-arm, open-label trial where PTSD symptoms were measured as a secondary outcome measure with the PTSD for DSM-5 (PCL-5) scale. Among 18 older long-term AIDS survivors, PTSD severity, as measured by PCL-5, decreased at weeks 1 and 2 post-psilocybin administration and at the 3-month follow-up. This study is limited by the significant range of standard deviation with overlapping scores when scores were measured at these timepoints. Furthermore, only 3 out of the 18 people met criteria for PTSD before being administered psilocybin, further limiting the applicability of this study (Anderson et al., 2020). However, several studies examining the role of psilocybin for the treatment of PTSD are underway, as seen in Table 2 (National Library of Medicine), including two studies examining psilocybin-assisted psychotherapy (PAP) for PTSD in veterans and one examining PAP for treatment-resistant PTSD.

**Table 2.** Studies registered on Clinicaltrials.gov examining psilocybin for PTSD.

Title	Status	Location	Last updated	Population	Intervention	Comparison	Outcome
Evaluation of psilocybin-assisted psychotherapy (PaP) for the treatment of post-traumatic stress disorder (PTSD) in military veterans	Not yet recruiting	Leatherhead, United Kingdom	May 25th 2023	8 military veterans	Psilocybin-assisted psychotherapy (2 dosing sessions followed by 12 sessions of cognitive processing therapy)	N/A	PTSD symptoms after 1 month follow-up
Psilocybin-assisted cognitive processing therapy for chronic PTSD	Not yet recruiting	Toronto, Canada	April 26th 2024	15 participants	Single dose of psilicybin 25 mg with 12 sessions of cognitive processing therapy	N/A	Clinician administered scales, self-reported questionnaires, data from a wearable device after 12 weeks
Psilocybin for the treatment of veterans with posttraumatic stress disorder	Recruiting	Columbus, USA	May 8th 2024	15 veterans age 21–64	Two doses of psilocybin with preparatory and post-therapy sessions	N/A	Not specified, follow-up visits will occur 1, and 2 weeks, as well as 1, 3, and 6 months after the final psilocybin session
A study of psilocybin for PTSD	Recruiting	Baltimore, United States	June 25th 2024	20 participants	Two doses of psilocybin and trauma-focused psychotherapy	Two doses of psilocybin and psychological support	Clinician and participant ratings of PTSD and mood symptoms pre and post the two psilocybin sessions, which are separated by 2 weeks

### Psilocybin as a treatment for PTSD: a mechanistic discussion

Although the exact mechanism of psilocybin and psilocin are still subjects of ongoing investigations, several mechanisms for the therapeutic benefits of psilocybin have been postulated, including its effect on emotional modulation, neurotransmitters, and neuroplasticity (Table 3).

**Table 3.** Possible mechanisms of psilocybin as a treatment for PTSD.

Mechanism	Description
Psychopharmacological	Reduces amygdala reactivity to negative stimuli
	Decreased threat response
	Increased amygdala responsiveness to positive stimuli
	Partial agonism of serotonin
Psychological	Loosening of defense mechanisms
	Release of unconscious materials
	Release of affective load
	Facilitation of transference
	Increased insight into the impact of trauma
	Facilitated synthesis of trauma into character

Psilocybin acts on many of the same areas of the brain affected in patients with PTSD. Psilocybin usage was associated with reduced amygdala reactivity to negative stimuli measured on fMRI (Kraehenmann et al., 2015), decreased threat response, as well as to increased amygdala responsiveness to positive emotional stimuli (Lowe et al., 2021).

Psilocybin also acts as a partial agonist of serotonin (5-HT), the same neurotransmitter targeted by conventional pharmacological treatment options for PTSD, such as Selective Serotonin Reuptake Inhibitors (SSRIs). Although psilocybin has been noted to act on multiple serotonin receptors such as 5-HT1A, 5-HT2B, 5-HT1D, and 5-HT2C (Dodd et al., 2022), it is thought to exert anxiolytic, antidepressant, and hallucinogenic effects mainly by acting as a partial agonist at the 5-HT2A receptor. 5-HT2A agonism has been associated with the enhancement of acquired fear extinction (Zhang and Stackman, 2015), positive mood, and attenuation of negative facial expressions (Kometer et al., 2012). As such, pharmacological approaches for PTSD include SSRIs and SNRIs. Though exact mechanisms are unclear, 5-HT2A agonism has also been associated with hallucinogenic effects via modulation of dopamine release in the striatum and the cortex (Raote et al., 2007). Psilocybin also increases dopamine, though not directly, within the mesolimbic dopaminergic pathway, which plays a significant role in the brain’s reward system (Lowe et al., 2021).

Psilocybin facilitates fear extinction and promotes neuroplasticity via neuritogenesis and spinogenesis both in vivo and in vitro, which may also contribute to antidepressant and anxiolytic effects (Krediet et al., 2020). These neuroplastic changes are thought to be mediated through tropomyosin receptor kinase B (TrkB), mammalian target of rapamycin (mTOR) and 5-HT2A signalling pathways (Dodd et al., 2022).

The mechanisms of therapeutic effects of psilocybin extend beyond psychopharmacology. The psychological mechanisms of psilocybin’s effects overlap with those of the other psychedelics. First, the importance of both “set” (personality, preparation, expectation, and intention of the user) and “setting” (physical, social, and cultural environment in which the therapy takes place) in determining the effects of psychedelics has been discussed in the literature (Hartogsohn, 2017). Reflecting this, patients undergoing psychedelic-assisted psychotherapy often undergo preparatory and integration therapy sessions in safe, comfortable, and trusting settings (Bird et al., 2021). In many studies, psilocybin use also often takes place with the aid of music, and participants are often advised to stay with and describe the subject matter which comes to mind (Davis et al., 2021; Goodwin et al., 2022; Von Rotz et al., 2023).

Second, psychedelics can assist with loosening of ego defenses, release of unconscious materials and affective load, increased insight into the impact of traumatic event on the creation of maladaptive ego defenses, facilitation of transference, and synthesizing traumatic experiences into a more flexible character structure (Buchborn et al., 2023). Since PTSD is associated with maladaptive defense mechanisms including projection, psychotic distortion, passive aggression, and dissociation (Vaillant, 2011), it stands to reason that psychedelics could help foster healing.

The Relaxed Beliefs Under Psychedelics and the Anarchic Brain (REBUS) draws upon both neuropsychiatry and psychoanalysis to explain the mechanism of psychedelics (Kelly et al., 2021). It proposes that psychedelics relax the typical constraints higher-order brain systems impose on emotion, cognition, and sensory perception through their action on the 5-HT2A receptor, allowing people to better modulate pathological fixed beliefs and opening them to considering information they would otherwise be biased to ignore or discount (Kelly et al., 2021). Psychedelics, thereby, could help people modulate firmly held maladaptive cognitive beliefs about their trauma, referred to as “stuck points” in Cognitive Processing Therapy, which is an evidence-based psychotherapy modality used for PTSD (Watkins et al., 2018).

## Conclusion

Psilocybin is well-poised to be a potential treatment option for PTSD, particularly for patients who cannot tolerate, access, or experience a subclinical improvement with conventional treatment options. Psilocybin has been shown to act on the same areas of the brain affected in patients with PTSD and acts on the same receptors as those targeted by conventional pharmacological agents. Psilocybin also plays a role in neuroplasticity and may weaken defence mechanisms, and as such, it is already being used in conjunction with psychotherapy. Further research is required to investigate the efficacy and safety of psilocybin for the treatment of PTSD.
